# The upper airway parameters: the potential diagnostic clues for congenital intrathoracic lesions

**DOI:** 10.1186/s12884-023-05599-1

**Published:** 2023-05-23

**Authors:** Shijing Song, Jingjing Wang, Li Wang, Chenxiao Hou, Qingqing Wu

**Affiliations:** 1 Department Ultrasound, Beijing Obstetrics and Gynecology Hospital, Capital Medical University, No.251 Yaojiayuan Road, Chaoyang District, 100026 Beijing, P. R. China; 2 Beijing Maternal and Child Health Care Hospital, Beijing, P. R. China

**Keywords:** Fetus, Airway, Congenital pulmonary airway malformation, Bronchopulmonary sequestration, Congenital diaphragmatic hernia

## Abstract

**Background:**

The diagnosis of congenital intrathoracic lesions still has limitations. The airway development was influenced by intrathoracic factors. Whether the diagnostic value of the upper airway parameters in congenital intrathoracic lesions has not been confirmed.

**Objectives:**

We aimed to compare fetal upper airway parameters between normal fetuses and fetuses with intrathoracic lesions, and we tried to verify its diagnostic value in intrathoracic lesions.

**Methods:**

This was an observational case–control study. In the control group, 77 women were screened at 20–24 weeks’ gestational age, 23 were screened at 24–28 weeks’ gestational age, and 27 were screened at 28–34 weeks’ gestational age. In the case group, 41 cases were enrolled (6 cases of intrathoracic bronchopulmonary sequestration, 22 of congenital pulmonary airway malformations, and 13 of congenital diaphragmatic hernia). Fetal upper airway parameters (tracheal width, the narrowest lumen width, and width of the subglottic cavity and laryngeal vestibule) were measured using ultrasound equipment. The correlations between fetal upper airway parameters and gestational age, and the differences in fetal upper airway parameters between cases and controls, were analyzed. The standardized airway paraments were acquired, and their potential diagnostic value for congenital intrathoracic lesions were analyzed.

**Results:**

The fetal upper airway parameters of both groups were positively correlated with the gestational age: The control group, tracheal width (*R*^2^ = 0.569, *p* < 0.001), narrowest lumen width (*R*^2^ = 0.429, *p* < 0.001), subglottic cavity width (*R*^2^ = 0.551, *p* < 0.001), laryngeal vestibule width (*R*^2^ = 0.349, *p* < 0.001). The case group (tracheal width *R*^2^ = 0.474, *p* < 0.001) narrowest lumen width (*R*^2^ = 0.425, *p* < 0.001), subglottic cavity width (*R*^2^ = 0.623, *p* < 0.001), laryngeal vestibule width (*R*^2^ = 0.347, *p* < 0.001). Fetal upper airway parameters of the cases group were smaller than those of the controls group. The tracheal width in fetuses with congenital diaphragmatic hernia was the smallest among the other case groups studied. The standardized tracheal width has the best diagnostic value for congenital intrathoracic lesions in the standardized airway paraments (the area under the ROC curve was 0.894), and has a high diagnostic value for congenital pulmonary airway malformations and congenital diaphragmatic hernia (the area under the ROC curve was 0.911 and 0.992, respectively).

**Conclusion:**

Fetal upper airway parameters differ between normal fetuses and fetuses with intrathoracic lesions, and might offer potential diagnostic clues for congenital intrathoracic lesions.

## Background

Diagnosis of congenital lung malformations has dramatically developed in recent years, but there are still some refinements. The small malformations of lung and the diaphragmatic hernia mainly composed by liver were easily misdiagnosed [1]. Biomechanics and lung luminal fluid, which often impaired by intrathoracic lesions, are key to fetal airway development [2–4]. Cilley et al. demonstrated that the absence of airway pressure impaired lung development, while the existence of airway pressure enhanced it owing to increased gene expression, such as hepatoma-derived growth factor, ribosomal protein S24, stathmin, and parathyroid hormone [5]. Fetal airway liquid is a major determinant of development and morphology of the fetal airway [6], given that the liquid acts as a medium for transmission of pressure. Fetal lung growth depends on the degree of distension created by the luminal liquid [7].

The trachea and bronchi are mainly composed of cartilage and smooth muscle; the development of those two components is influenced by each other [8]. Transmural pressure can promote the growth of smooth muscles in the airway [4, 9]. Further, the epithelial development of the airway wall is influenced by pressures such as shear stress [10].

In fetuses with intrathoracic lesions, the pulmonary volumes and capacities are known to be decreased [11]. The liquid present in the airway lumen and that secreted by pulmonary epithelium are both decreased, thus mitigating the pressure and expansion effect of the liquid. A reduction in the liquid also diminishes the effect of a mechanical stimulus, such as shear stress and transmural pressure. Therefore, we hypothesized that thoracic lesions might affect the development of the trachea, and consequently, the airway parameters might differ between normal fetuses and those with intra-thoracic lesions.

Prenatal ultrasonic imaging and experimental models have provided a comprehensive understanding of intrathoracic lesions [12]. Fetal ultrasonography allows the identification of fetal malformations [13] as well as examination of the development of fetal airways [14]. Since measurements are affected by different sample preparations, there are several limitations to using specimens for airway studies [15, 16]. Therefore, ultrasonography is thought to be the preferred option for studying living fetal trachea [17]. In this study, we aimed to analyze the fetal upper airway parameters using ultrasound, and explore its diagnostic value for congenital intrathoracic lesions.

## Methods

This study was designed to explore the diagnostic value of fetal airway paraments for congenital intrathoracic lesions, and was a single-center, prospective, case-controlled, observational study performed between December 2020 to June 2021 at Beijing Obstetrics and Gynecology Hospital, Beijing, China. This study was approved by the Institutional Review Boards/Ethics Committees of Beijing Obstetrics and Gynecology Hospital. The inclusion criteria for the control group were as follows: a normal singleton fetus with known gestational age (by last menstrual period date or by early ultrasound exam) and followed up through September 2021 without any gross malformations. The inclusion criteria for cases were as follows: a singleton fetus with an intrathoracic lesion with no associated genetic or major anomalies. The following were the exclusion criteria: pregnant women with discomfort who could not tolerate sonography examinations; and/or the obtained images were not satisfactory for maternal and/or fetal reasons. One case in the control group was excluded for the poor imaging quality (due to the thick fat layer of abdominal wall in the gravida) and three cases in the case group were excluded for losing of follow-up. Finally, 127 controls were randomly selected and recruited. Of these, 77 were screened at 20–24 weeks’ gestational age, 23 were screened at 24–28 weeks’ gestational age, and 27 were screened at 28–34 weeks’ gestational age. Forty-one cases were enrolled (22 cases of congenital pulmonary airway malformations (CPAMs), 6 cases of bronchopulmonary sequestration (BPS), and 13 cases of congenital diaphragmatic hernia (CDH). Among these, 14 were screened at 20–24 weeks’ gestational age, 16 were screened at 24–28 weeks’ gestational age, and 11 were screened at 28–34 weeks’ gestational age. CPAMs and BPS were confirmed by newborn computed tomography, newborn surgery. CDH was confirmed by prenatal magnetic resonance imaging, newborn surgery, or fetal autopsy. Examinations were performed by two certified sonographers (L.W. and Q.W.) at our center. The measurements were obtained using ultrasound equipment (WS80A, Samsung Medison Co., Ltd., Seoul, South Korea) with a CV1-8A probe. All pregnant women signed informed consent forms prior to study enrollment. The upper airway parameters measured included: tracheal width (TW), subglottic cavity width (SW), narrowest lumen width (NW), and laryngeal vestibule width (LW) [18]. All upper airway parameters were measured in all cases. The standardized airway paraments (SAPs) were used to rule out the effect of gestational week. Specifically, divide airway paraments by gestational week to get the standardized airway paraments.

**Fig. 1:**
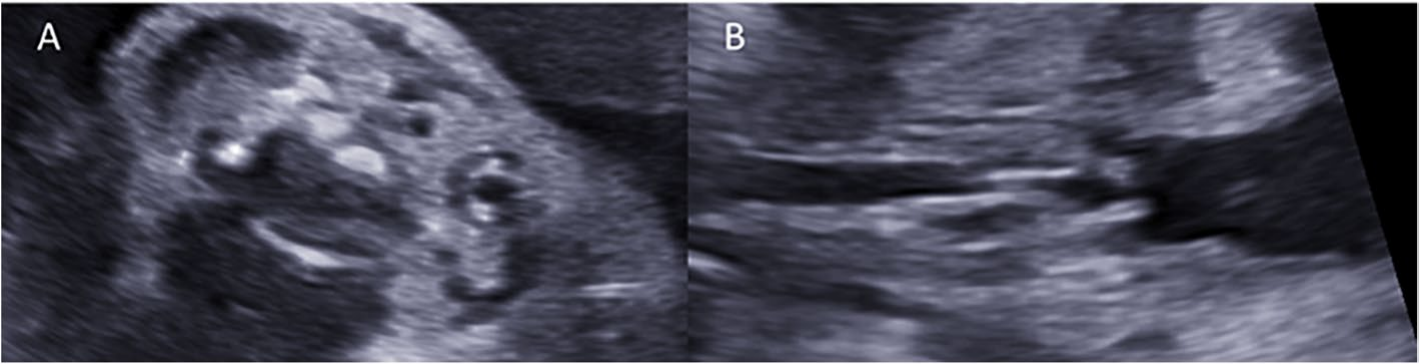
**A** The transection plane, **B** The coronal plane

In previous studies, the measurements have varied with the methods used for assessment of tracheal parameters [19]. In the current study, we took the following simple approach to standardize measurements. At first, we get the coronal section of the trachea. We display the transverse plane of the trachea, keeping the insonation perpendicular to the trachea. Then, rotate the probe 90° to display the coronal plane (Fig. 1). To avoid Intraluminal pressure influence, the state that the fetus without body movement and respiratory movement was chosen.

### Tracheal width

**Fig. 2:**
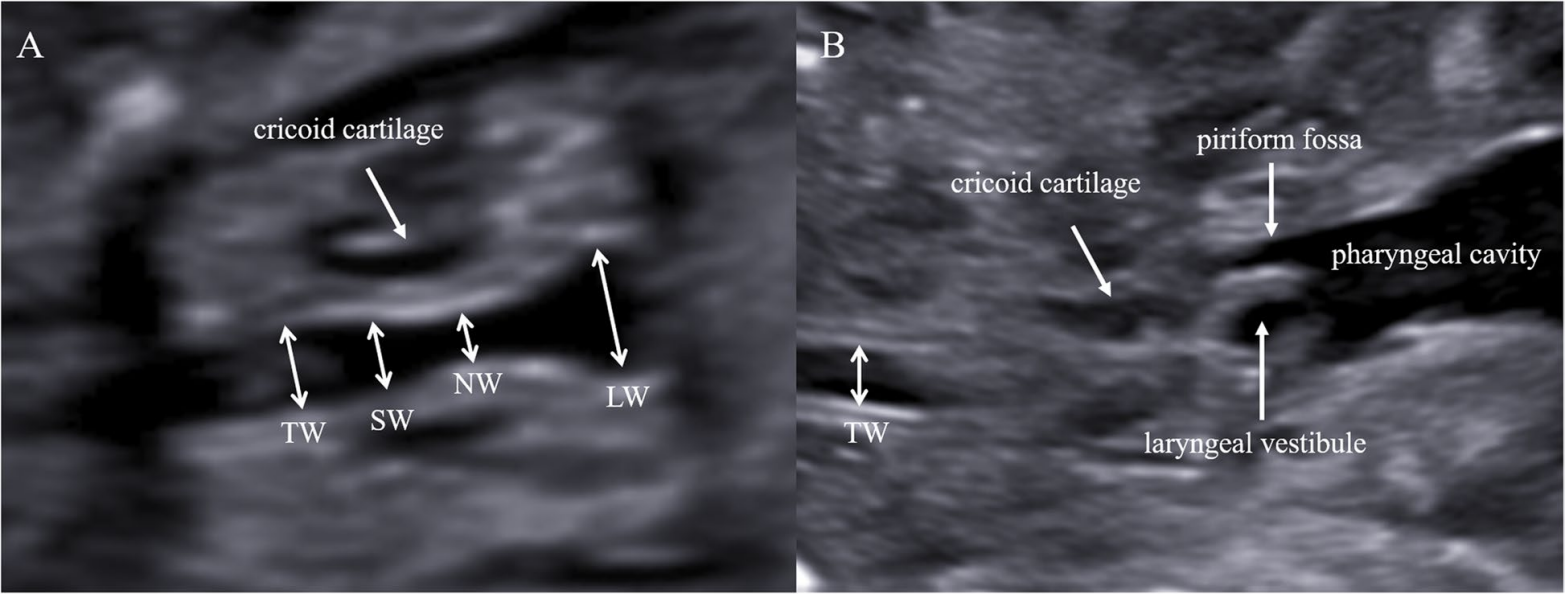
**A** The opening of the laryngeal lumen, **B** The closing of the laryngeal lumen. (TW: tracheal width, SW: width of subglottic cavity, NW: the narrowest lumen width, LW: width of laryngeal vestibule)

The tracheal ring is composed of a “C” type cartilage ring, with the free ends of cartilages at the posterior border being bridged by smooth muscle [20]. On the coronal plane, the distance between the hyperechoic lines at the edge of the tracheal lumen was defined as the tracheal width. The coronal plane was continuously scanned to determine the plane with the largest tracheal width. Next, the tracheal width was measured 0.5–1 cm distal to the cricoid cartilage, making sure that the trachea wall was clearly displayed, both sides of the wall were hyperechoic, and the body of tracheal cartilage was anechoic. However, the opening and closing of the pharyngeal cavity displaces the proximal tracheal tissue. This interference can be avoided at about 0.5 cm from the lower edge of cricoid cartilage (Fig. 2). When the distance from the lower edge of cricoid cartilage exceeds 1 cm, the rotation of the fetal head and neck hinders the presentation of the standard coronal plane.

### Laryngeal vestibule width

Lateral walls of the laryngeal vestibule are composed of upright aryepiglottic folds [21]. The distance between the inner border of the aryepiglottic folds was defined as the LW. The LW was measured when the thickness and length of cricoid cartilage were equal on both sides. In this plane (Fig. 2), the area of the piriformis fossa was also equal on both sides.

### Subglottic cavity width

The shape of subglottic cavity was similar to a cone, and the measuring position of SW may be different from that of forward and backward, the upward and downward will also make measurement bias. Therefore, we chose the coronal plane at the midpoint of cricoid cartilage for standardized measurement.

### Narrowest lumen width

The NW was recorded close to the level of the upper border of the cricoid cartilage [15].

Artifacts such as the partial volume effect may affect the accuracy of image. Accordingly, using an angle of insonation perpendicular to the airway wall is important to avoid artifacts [22]. Each observer made measurements independently in this study.

Data was analyzed with SPSS® software version 26.0 (IBM Corp., Armonk, NY, USA). The Kolmogorov–Smirnov test was performed on all measured parameters to assess the normality of distribution. Non-parametric tests were performed when the data did not follow a normal distribution and when the variance was not homogeneous, and result was presented as median(rage). The correlation between upper airway parameters and gestational age was analyzed. The fitted growth curves of the upper airway parameters were obtained. Receiver operating characteristic (ROC) curves were plotted to assess the diagnostic performance of SAPs on congenital intrathoracic lesions. Select SAP with the best diagnostic performance to verify its potential diagnostic value for CPAMs and CDH. Independent samples *t*-tests were applied to calculate group differences. To calculate inter-observer variation, differences between the repeated measurements were evaluated by the intraclass correlation coefficient (ICC). Twenty fetuses were randomly selected for ICC calculation and measured by two observers blinded to each other’s measurements.

Two tracheal gross specimens of fetuses with the same gestation age were assessed, one fetus with CDH and pulmonary dysplasia ((gestational weeks (GW): 23W3D, bi-parietal diameter (BPD) = 5.8 cm, head circumference (HC) = 20.7 cm, abdominal circumference (AC) = 20.3 cm, and femur length (FL) = 3.8 cm)), and another fetus with a single ventricle of the heart and normal lung development (GW: 23W4D, BPD = 6 cm, HC = 22.5 cm, AC = 18.9 cm, and FL = 3.9 cm).

## Results

The age (*p* = 0.098), weight (*p* = 0.007), BMI (*p* = 0.001) data of case group and age (*p* = 0.019), height (*p* = 0.028), weight (*p* = 0.002) data did not fit the normality test. Thus, nonparametric tests were applied. No statistically significant difference in age, height, weight, BMI data was noted between the two groups (*p* = 0.073, 0.194, 0.504, 0.985, respectively). The gestational week distribution of the three diseases was not a normal distribution. Applying the nonparametric multiple independent sample test, the progressive significance *p* = 0.889 is obtained, which proves that there is no statistical difference between the gestational week of the three diseases.

Airway paraments of the two groups with different gestational ages are shown in Table 1 (unit of TW, SW, NW, LW is millimeter). The upper airway parameters of the controls were expressed by the functions: TW = 0.30 + 0.1 × GW (*R*^2^ = 0.569, *p* < 0.001). NW = -0.3 + 0.07 × GW (*R*^2^ = 0.429, *p* < 0.001), SW = -0.4 + 0.1 × GW (*R*^2^ = 0.551, *p* < 0.001), LW = 0.5 + 0.1 × GW (*R*^2^ = 0.349, *p* < 0.001). In addition, the residual plot indicated a good fit of linear regression in control group. For the cases, TW *R*^2^ = 0.474, *p* < 0.001, NW *R*^2^ = 0.425, *p* < 0.001, SW *R*^2^ = 0.623, *p* < 0.001, LW *R*^2^ = 0.347, *p* < 0.001. However, normal distribution of residuals in case group was violated, so the regression equation for upper airway parameters could not be built.

**Table 1:** Airway paraments of the control and the case groups with different gestational ages

			Control group			Case group	
GW		N	Means	Standard deviation	N	Means	Standard deviation
**20-24**	TW	76	2.608	(0.320)	14	1.786	(0.313)
	SW	76	1.839	(0.240)	14	1.386	(0.228)
	NW	76	1.385	(0.206)	14	1.114	(0.238)
	LW	76	2.750	(0.447)	14	2.321	(0.473)
**24-28**	TW	25	2.956	(0.268)	16	2.338	(0.454)
	SW	25	2.200	(0.367)	16	1.794	(0.307)
	NW	25	1.672	(0.256)	16	1.494	(0.347)
	LW	25	3.088	(0.450)	16	2.744	(0.465)
**28-34**	TW	27	3.489	(0.465)	11	2.800	(0.387)
	SW	27	2.630	(0.536)	11	2.327	(0.410)
	NW	27	1.970	(0.441)	11	1.845	(0.408)
	LW	27	3.596	(0.653)	11	3.236	(0.484)

*TW* tracheal width, *SW* width of subglottic cavity, *NW* the narrowest lumen width, *LW* width of laryngeal vestibule, GW, gestational weeks. The unit of width is millimeter, the unit of GW is week

**Fig. 3:**
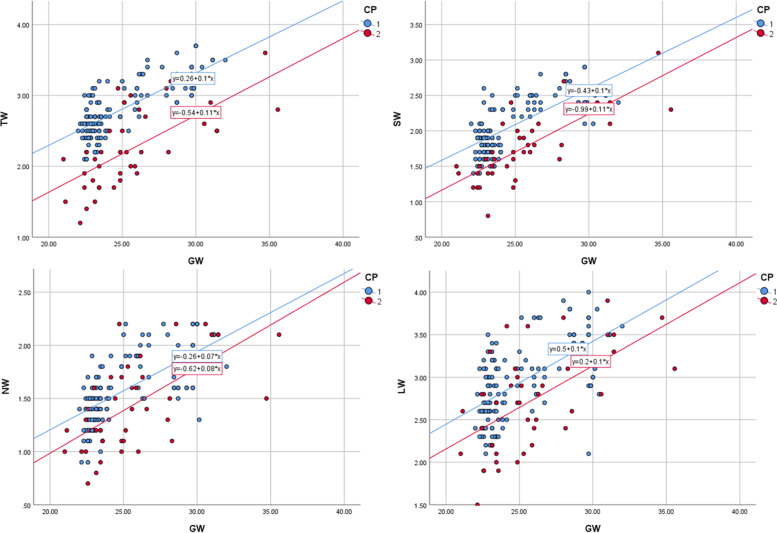
Fetal upper airway parameters of two groups increased with gestational age. (CP1: control group, CP2: case group, TW: tracheal width, SW: width of subglottic cavity, NW: the narrowest lumen width, LW: width of laryngeal vestibule, GW, gestational weeks. The unit of width is millimeter, the unit of GW is week)

**Fig. 4:**
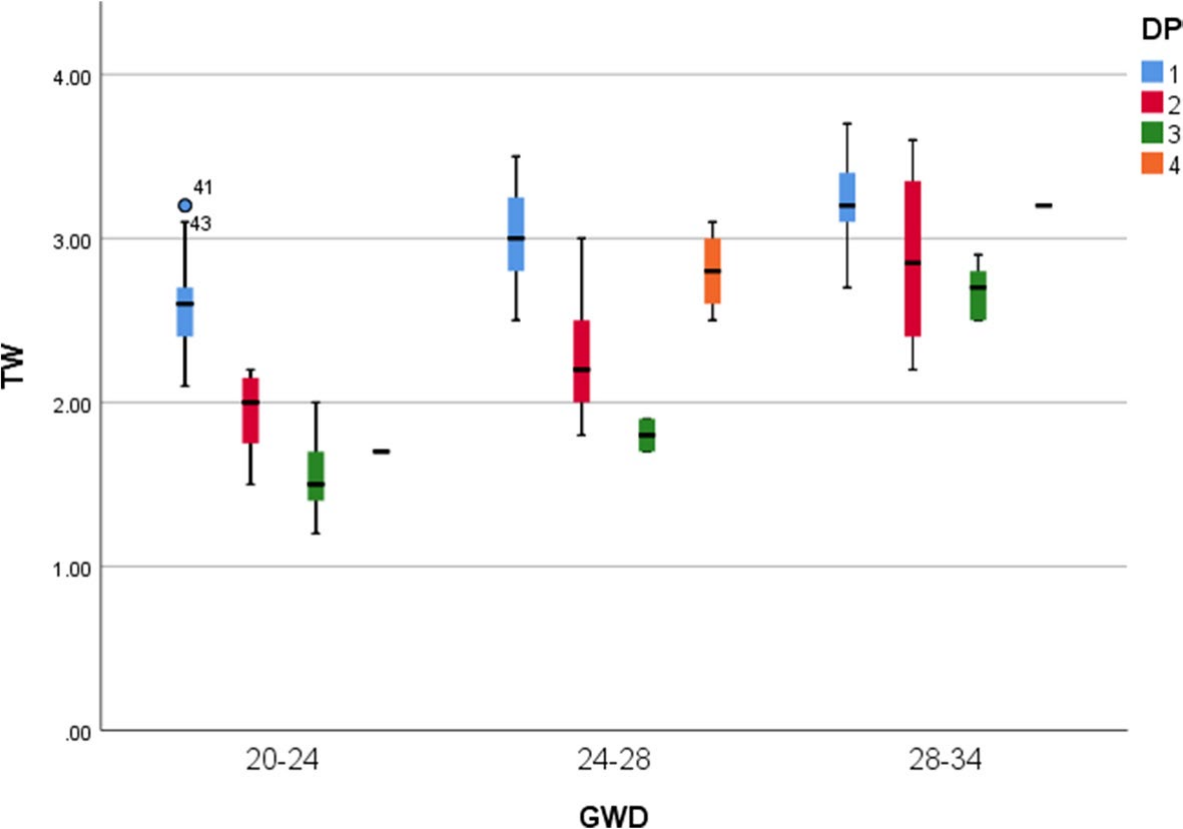
Width of trachea in CDH was the least compared to other case groups. (DP1: control group, DP2: CPAMs group, DP3: CDH group, DP4: BPS group, GWD: gestational weeks, dots and asterixis: outliers. The unit of width is millimeter, the unit of GWD is week)

**Fig. 5:**
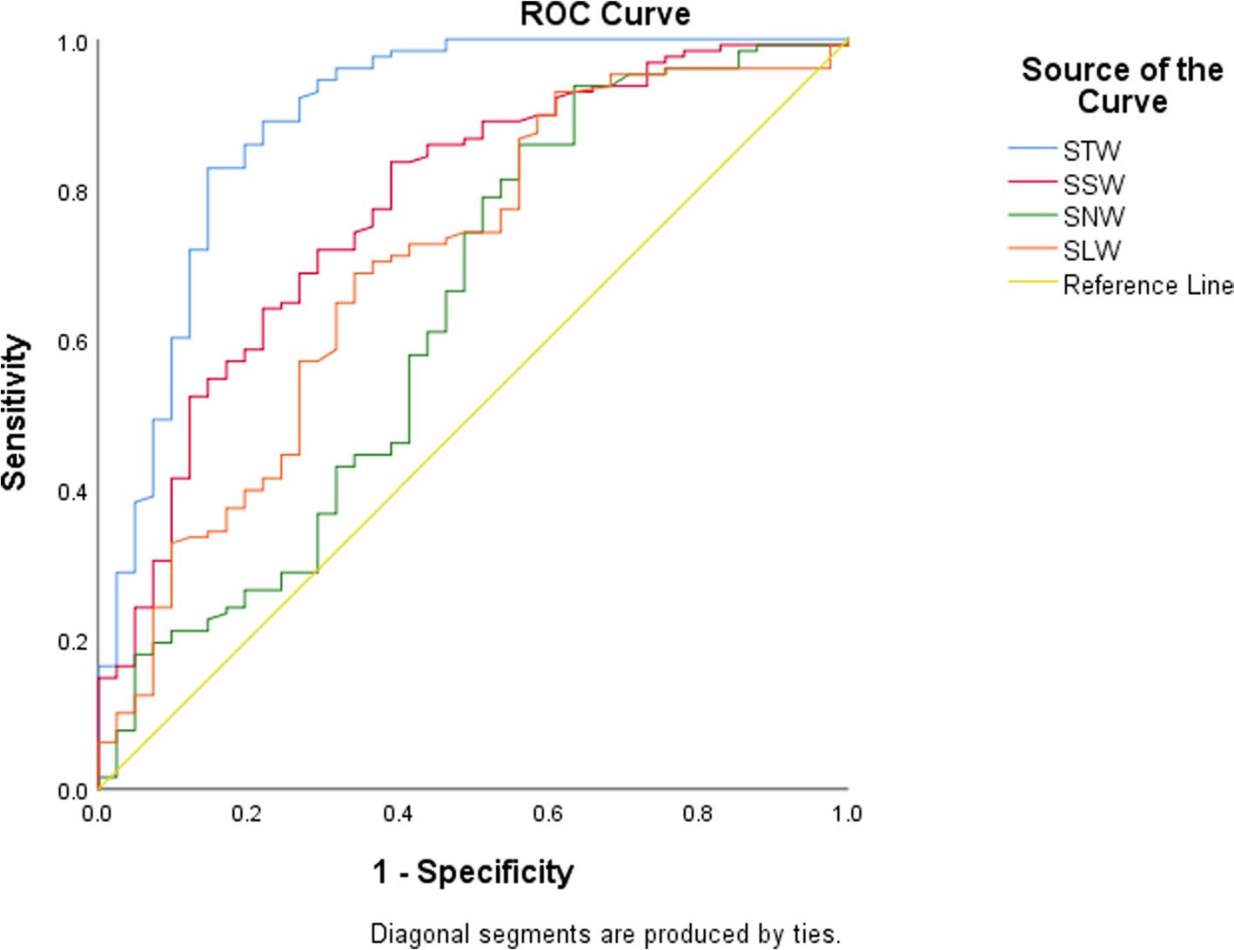
The Receiver operating characteristic curves of the SAPs in congenital intrathoracic lesions. STW AUC: 0.894, SSW: 0.776, SNW: 0.632, SLW: 0.697. (STW: standardized tracheal width, SSW: standardized width of subglottic cavity, SNW: standardized width of the narrowest lumen, SLW: standardized width of laryngeal vestibule, AUC: area under the curve)

**Fig. 6:**
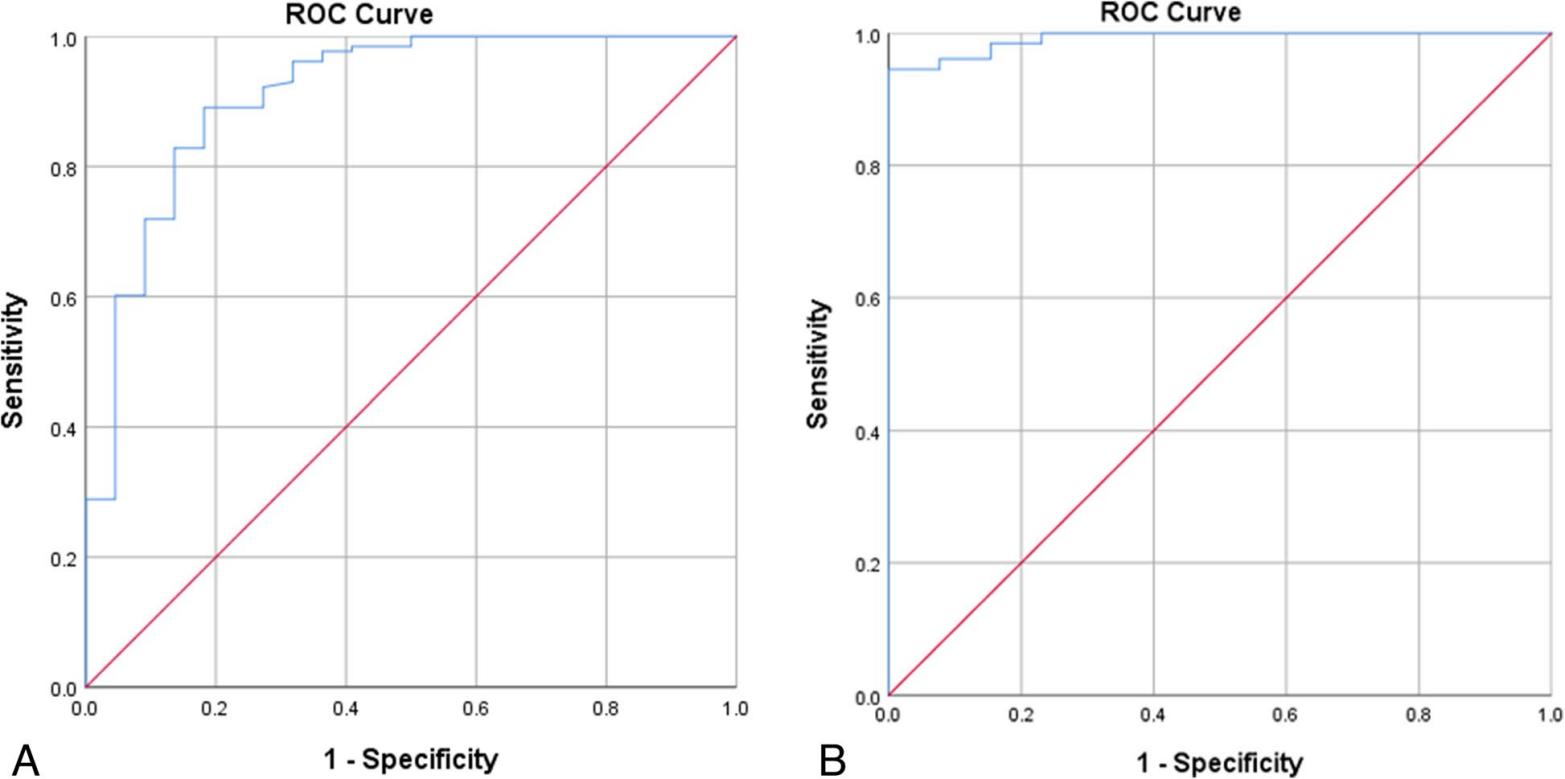
The ROC curves of the STW in CPAM (**A**) and CDH (**B**). AUC is 0.911 and 0.992, respectively. (STW: standardized tracheal width, AUC: area under the curve.)

The upper airway parameters in the cases were significantly smaller than those in controls (*p* < 0.01). The gestational age of the two groups was not significantly different (*p* = 0.61). The gestational week distribution of the three diseases was not a normal distribution. Applying the nonparametric multiple independent sample test, the progressive significance *p* = 0.889 is obtained, which proves that there is no statistical difference between the gestational week of the three diseases. The TW of three diseases was not fit normal distribution, and nonparametric test was applied. Upper airway paraments in the three diseases were not consistent (*p* = 0.043). TW of CPAMs = 2.200 (1.500–3.600), TW of CDH = 2.000 (1.200–2.900), TW of BPS = 2.800 (1.700–3.200). The upper airway parameters of the cases and controls increased with gestational age and had a strong positive correlation with the gestational age (Fig. 3). Compared with other case groups, the width of the trachea in the CDH group was smaller and more strongly correlated with the gestational age (Fig. 4). The SAPs of the two groups were acquired (control group: STW = 11.3 ± 1.3, SSW = 8.1 ± 1.48, SNW = 6.1 ± 1.26, SLW = 11.9 ± 1.9; case group: STW = 8.7 ± 1.68, SSW = 6.8 ± 1.26, SNW = 5.6 ± 1.35, SLW = 10.6 ± 1.9). The areas under the ROC curve were acquired (STW = 0.894 95% CI: 0.829–0.960, SSW = 0.776 95% CI: 0.693–0.859, SNW = 0.632 95% CI: 0.54–0.740, and SLW = 0.697 95% CI: 0.602–0.793) (Fig. 5). The STW with the best diagnostic performance, the cut-off value was 1.04 (sensibility: 0.828, specificity: 0.854). The area under the ROC curve was 0.911 (CI: 95%, 0.838–0.985) for CPAMs and 0.992 (CI: 95%, 0.980–1.000) for CDH, respectively (Fig. 6). The cut-off value was 1.00 for CPAMs (sensibility: 0.891, specificity: 0.818) and 0.95 for CDH (sensibility: 0.945, specificity: 1), respectively.

The ICC values demonstrated high inter-observer reproducibility for TW (ICC = 0.958), SW (ICC = 0.966), NW (ICC = 0.805), and LW (ICC = 0.848).

**Fig. 7:**
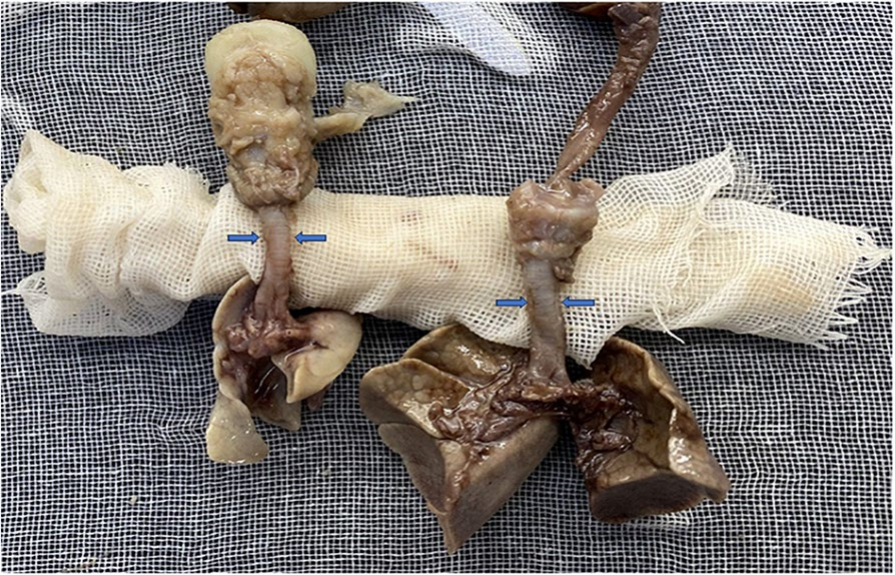
At the same gestational age, the trachea of the fetus with a normally developed lung (right) was wider than that of a fetus with pulmonary dysplasia caused by CDH (left). Blue arrows show the width of trachea

The gross specimens of the lungs are shown in Fig. 7. The trachea of the fetus with a single ventricle but normal lung development was wider than that of the fetus with pulmonary dysplasia caused by CDH.

## Discussion

### Principal findings

The airway paraments in fetus with intrathoracic lesion was smaller than normal fetus. The standard airway paraments has potential diagnostic value for intrathoracic lesion in 20–34 gestational week fetuses.

We chose to measure the width of the airway for the following two reasons. First, the change in the tracheal width appears to be more pronounced during pregnancy. Szpinda et al. found in fetal specimens that the trachea was almost circular at 14–18 weeks’ gestational age and more D-shaped at 21–25 weeks’ gestational age [16], indicating that the change in width is more obvious than change in length in the horizontal plane. Cartilage is the main component of the tracheal wall [8]. On ultrasonography, fetal cartilage has an anechoic body and a hyperechoic edge [23]. The diameter between the hyperechoic lines of the inner edges of both sides of the tracheal wall may reflect the true internal diameter. In contrast, the posterior wall of trachea is composed of soft tissue which might merge with the surrounding tissue on sonography, thus affecting the accuracy of inner-diameter measurements.

### Results in the Context of What is Known

The tracheal width in this study was similar to the results of Kalache et al. (from 2.14 ± 0.40 mm to 4.32 ± 0.89 mm between 20–38 weeks gestational age) [24], and slightly different from those of Richards et al. (from 2.4 mm at 18 weeks’ gestational age to 4.6 mm at 38 weeks’ gestational age) and Cao et al. (from 1.8 mm at 20 weeks’ gestational age to 4.7 mm at 40 weeks’ gestational age) [14, 25]. For the Cao’s study, we speculate that the reasons may be the different measuring positions. The different between our result and Richard’s might because the development of ultrasound technique during the nearly 3 decades. Thus, the standardized measurement method was needed.

During the embryonic phase, fetal lungs originate from two outpouchings of the foregut that eventually form the trachea and esophagus [2]. And then the airway parament was developed with gestational age [19] as our study proved. Consistent with previous findings, this study demonstrated a correlation between gestational age and airway paraments in the control group [14, 24, 25]. In contrast to the study by Szpinda et al., our correlation was weaker (*R*^2^ = 0.57 VS *R*^2^ = 0.81) [16]. This may have been a result of two observers performing the measurements in our study, which may have produced observer bias to some extent, though there was no significant interobserver difference.

The upper airway paraments were positively correlated with the gestational age in the case group too, and in the control group were larger than those in the case group. These results validate our hypothesis that intrathoracic lesions might impede airway development. Biomechanics and liquid in the lungs are important determinants of fetal lung development [6, 26]. Biomechanics are mainly influenced by fetal breathing motions and transpulmonary pressures created by the lung liquid [27, 28]. The lung liquid, produced by lung epithelial cells, serves as the medium for transmission of pressure and helps maintain fetal lung expansion [2]. At the same time, fetal lung liquid production and secretion are enhanced by intra-amniotic pressure and fetal breathing motions [26]. Fetal breath movements enhance lung growth and airway expansion [29]. Intrathoracic lesions in the fetus can lead to lung hypoplasia [2]. Both CPAMs and BPS are associated with decreased fetal lung volume and reduced fetal lung liquid secretion. The intrathoracic pressure is increased while the transmural pressure of the trachea is decreased during exhalation. CDH is caused by insufficiency of the diaphragm, potentially leading to pulmonary hypoplasia [30], which can impede movement of the diaphragm and decrease the fetal lung volume. Pressure caused by diaphragmatic movement and lung liquid secretion is also decreased, which may explain why the width of trachea in the CDH group was minimal.

The correlations of gestational age with TW and SW were stronger than those with NW and LW. This may be attributed to the mechanical support provided by the cartilage to the wall of the trachea and subglottal cavities. Second, the movement of the wall of the laryngeal vestibule and the narrowest lumen might affect stability during measurements.

The upper airway parameters in the CDH group were better correlated with gestational age compared to those with other case groups, and were found to be minimal in all the other groups. This may be because the volume of CPAMs will decrease with gestational age, and the effect on the lung will also decrease [31]. The dispersion of airway paraments of case group was dispersion. We speculate that some cavities of CPAMs were communicated with the normal airway, so that the amount of fluid flowing through the trachea did not decrease.

### Clinical and research implications

The detecting of the small size congenital intrathoracic lesions still needs further improvement [1]. CDH may be hard to diagnose because the herniated liver and lung have the same sonographic characteristics [32]. The SAP has proved its potential diagnostic value for congenital intrathoracic lesions and CDH in this study, without intrathoracic lesions detecting limitations.

The tracheal diameter in fetuses with laryngeal atresia was found to be significantly higher than that in normal fetuses [24]. In this study, we observed that the upper airway parameters of fetuses with intrathoracic lesions were smaller than those of normal fetuses. Therefore, when applying the transtracheal management for diagnosis and treatment, such as for endoscopic tracheal occlusion on fetuses and neonates, instrument selection should be performed carefully.

### Strengths and limitations

The upper airway paraments might provide a novel method to diagnose congenital intrathoracic lesions without intrathoracic detecting limitations. We speculate that, according to the principle of tracheal development, the upper airway paraments might be prognostic mark which linked to lung functions.

There were some limitations to the sonographic assessment of the severity of fetal intrathoracic lesions. First, fetal position and thorax may impede the proper measurement of lesion diameters. Second, the shape of lesion is always irregular, therefore, the results obtained by the above formula may differ from the true volume. Therefore, structural characteristics outside of the thoracic trachea might provide new diagnostic clues. Relatively speaking, airway examinations, especially that of the cervical trachea, are less affected by fetal position and thoracic parameters. The CPAMs volume ratio (CVR) is a measurement of the tumor normalized for gestational age. The shape of the CPAMs is assumed to be roughly elliptical [33]. Similar to CPAMs, BPS are also characterized by non-functioning lung tissue, may form hybrid lesions with CPAMs [1], and share the same prognostic factors with CPAMs [34].

The existence of CDH interferes with normal fetal lung development in intrauterine life, leading to decreased bronchiolar branching, small lung size, and hypoplasia [32]. The prenatal diagnosis of CDH is based on ultrasound imaging. As prognostic predictors for CDH, the lung-to-head circumference ratio (LHR) and observed/expected LHR are still controversial and have some limitations [32, 35].

Lung function is not only linked to its volume, but also to its biomechanics [36]. Volume parameters can only reflect the change in lung volume. Meanwhile, the upper airway parameters, influenced by biomechanics and lung liquid, might reflect true lung function. Some scholars once believed that the LHR only has prognostic value in left-sided CDH [32], however, recent research indicates that LHR can also be used for right diaphragmatic hernia [37]. Meanwhile, the upper airway parameters may be associated with overall lung development, thereby possessing prognostic value for potentially all types of CDH.

In this study, the airway paraments were related linearly to gestational age during 20–34 gestational week. The SAP was used to rule out the effect of gestational week, and as a diagnostic index for congenital intrathoracic lesions. This study demonstrated that the airway paraments, especially the STW, have a high diagnostic accuracy for congenital intrathoracic lesions. But there are still some limitations. Firstly, as the R-squared is not high, the linear relationship between upper airway parameters and gestation age is not strong. So, there is limitation in using SAP to correct gestational age. Secondly, the case group has been confirmed by ultrasound, those intrathoracic lesions cannot be diagnosed by ultrasound are missed. What is more, successful rate of the measurement of the airway and the size of intrathoracic lesions in those cases were not presented. It is not known whether these lesions were small.

We were unable to assess the change in upper airway parameters in many kinds of diseases as the sample size was not sufficient to allow such an analysis. Different stages of lung development are influenced by different factors [2], pressure and liquid may play different roles in different stages of lung development. Therefore, further investigation on the influence of different pathologies, gestational age, and volume change of lesions on airway growth is warranted. To this effect, we are in the process of collecting more cases of intrathoracic lesions to analyze the correlation between the upper airway parameters and CVR and LHR. In our future study, we hope to assess the value of upper airway parameters in the prognostication of intrathoracic lesions. Considering that the volume of lesions decreases in approximately 15% of CPAMs and 68% of BPS [11], the airway development may change accordingly. However, due to the small sample size for sequential observation in this study, the correlation between the upper airway parameters and change in size of lesions needs to be studied further.

## Conclusions

The airway diameters increased with gestational age. In fetuses with intrathoracic lesions, the upper airway diameters were found to be decreased compared to those in normal fetuses. Fetuses with CDH had the smallest tracheal diameters among other case groups. The upper airway parameters, especially the standardized tracheal width, are expected to provide novel potential diagnostic clues for congenital intrathoracic lesions.

## Data Availability

The datasets used and/or analyzed during the current study are available from the corresponding author on reasonable request.

## Abbreviations

CPAMs: Congenital pulmonary airway malformations
BPS: Bronchopulmonary sequestration
CDH: Congenital diaphragmatic hernia
TW: Tracheal width
SW: Subglottic cavity width
NW: Narrowest lumen width
LW: Laryngeal vestibule width
SAPs: Standardized airway paraments
ROC: Receiver operating characteristic
ICC: Intraclass correlation coefficient
GW: Gestational weeks
BPD: Bi-parietal diameter
HC: Head circumference
AC: Abdominal circumference
FL: Femur length
